# Lobar pattern of microbleeds on susceptibility-weighted magnetic resonance imaging

**DOI:** 10.4103/0972-2327.70885

**Published:** 2010

**Authors:** N. Shobha, Eric E. Smith

**Affiliations:** 1 Calgary Stroke Program, Department of Clinical Neurosciences, University of Calgary, Calgary, Alberta, Canada; 2 Calgary Stroke Program, Department of Clinical Neurosciences; Hotchkiss Brain Institute, University of Calgary, Calgary, Alberta, Canada

**Keywords:** Amyloid angiopathy, microbleeds, susceptibility-weighted imaging

**Figure 1:**
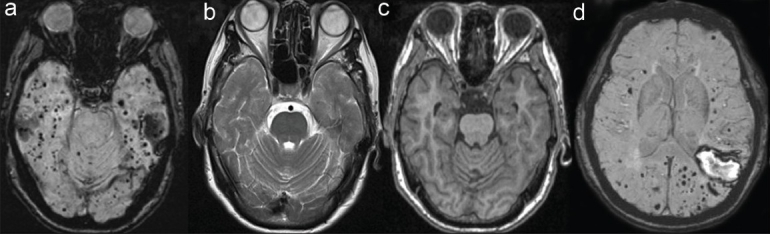
(a) Multiple hypointense lesions seen on MR - SWI susceptibility-weighted imaging involving the lobes of the cerebrum and cerebellum suggestive of microbleeds. (b and c) (T2WI) and (T1WI), respectively, demonstrate no hyperintense lesions. (d) The absence of microbleeds in the basal ganglia and deep white matter and an acute lobar macrobleed in the left temporoparietal region on MR - SWI

A 75 year old lady presented with progressive speech difficulty of one month duration with no history of motor weakness, ataxia, sensory, or visual symptoms. She did not have history of fever, headache, or seizures. She had no history of hypertension, diabetes mellitus, coronary artery disease, atrial fibrillation, or smoking. Details of her cognitive status were not known as she lived alone. Examination revealed a conscious and alert woman with fluent aphasia and no visual or sensorimotor deficits or meningeal signs. A detailed mental status examination was not possible due to aphasia. Her vitals were stable with a blood pressure of 108/70 mm Hg. Systemic examination was unremarkable. An MRI (Magnetic Resonance Imaging) of her brain showed this picture [Figure 1a–1d]. She was diagnosed to have cerebral amyloid angiopathy (CAA).

CAA is caused by progressive accumulation of congophilic amyloid in the walls of small- and medium-sized cerebral arteries with subsequent degenerative vascular changes. One of the most recognized complications of CAA is spontaneous, intracerebral hemorrhage, involving the cortex, subcortical white matter (“lobar hemorrhage”), which is recurrent. It can also present with dementia, stereotyped focal neurological symptoms or the patients can be asymptomatic.

The MRI of our patient showed numerous microbleeds involving the lobes of the cerebrum and cerebellum, sparing the basal ganglia and the deep white matter and an acute lobar macrobleed in the left temporoparietal region.

Cerebral microbleeds (CMBs) are small (<5.7 mm) MRI signal voids indicative of perivascular collection of hemosiderin deposits that are foci of past hemorrhages. There are several recommended criteria for CMBs.[1] Microbleeds are round or ovoid lesions hypointense on T2* gradient recalled echo (GRE) and susceptibility weighted imaging (SWI) and devoid of signal hyperintensity on T1 and T2 weighted imaging. At least half of the lesion is surrounded by brain parenchyma. An acute macrobleed on SWI sequence appears hyperintense in the center and hypointense in the periphery as deoxygenation occurs first at the periphery of the hematoma and progresses toward the center. This pattern appears because intrahematoma oxygen tension is lowest in the periphery, where red cells are adjacent to oxygen-starved tissue, and highest in the center, because red cells do not use oxygen for their metabolism.[2] Susceptibility effect is present when iron atoms are compartmentalized within the red cell membrane, causing magnetic field inhomogeneity, with resulting loss of phase coherence and selective shortening of the T2 relaxation time. After degradation of red cell membranes, the iron becomes more homogenously distributed, and this effect is nullified.[2]

SWI is a recently developed MR sequence where the image is generated from differences in susceptibility effect between tissues, and is just entering clinical practice. SWI is more sensitive than the commonly used T2* gradient recalled echo (GRE) for detection of microbleeds.[3] Based on the Boston criteria,[4] our patient was classified as probable CAA.

In population-based studies, CAA prevalence was consistently higher in the demented as compared to the non-demented subjects.[5] This supports a significant role for CAA in the pathogenesis of dementia. As the life expectancy of the Indian population increases, the incidence of CAA and dementia are expected to surge making it necessary for physicians to familiarize themselves with the clinical and imaging picture of CAA.
